# Sequential Bilateral Total Hip Arthroplasty Through a Minimally Invasive Anterior Approach is Safe to Perform

**DOI:** 10.2174/1874325001711011417

**Published:** 2017-11-30

**Authors:** Gerasimos Petridis, Martin Nolde

**Affiliations:** Orthopraxx Center for Orthopaedic Surgery, Munich, Germany

**Keywords:** Osteoarthritis, Hip, Minimally invasive surgical procedure, Sequential bilateral total hip arthroplasty, Outcome study, Harriship score, anterior approach

## Abstract

**Background:**

Sequential bilateral total hip arthroplasty (THA) has the potential advantages of a single operative intervention with a single hospital stay, alongside reduced costs and total rehabilitation times. Its use has been limited, however, by a theoretical increase in perioperative complications.

**Objective:**

The purpose of this study was to assess functional outcomes and complications in patients undergoing sequential bilateral THA performed using anterior minimally invasive surgery (AMIS). We hypothesized that sequential bilateral THA yields favorable clinical outcome and is safe to perform.

**Methods:**

Two surgical centres conducted a retrospective observational analysis of 130 patients (77 females) with a mean age of 57 (range, 35-77) years, all of whom were treated by one surgeon and followed up for 24 months.

**Results:**

The mean length of hospital stay length was 8.4 (range, 6–18) days. The mean operative time was 162 (range, 92–185) minutes, the mean intraoperative blood loss was 499.1ml, and the mean preoperative and postoperative hemoglobin levels were 14.3 g/dl and 11.3 g/dl, respectively. No perioperative complications or deaths were recorded. The Harris Hip Score (HHS) improved from 44.5 ±13.7 preoperatively to 98.9 ± 1.0 at final follow-up. Also the High Activity Arthroplasty Score (HAAS) and the Questions on Life Satisfaction (FLZ) score improved significantly.

**Conclusion:**

This retrospective analysis suggests that, in selected patients, sequential bilateral THA *via* an anterior minimally invasive approach appears to be a valid alternative to two-stage bilateral THA. Further studies are warranted.

## 1 INTRODUCTION

Sequential bilateral total hip arthroplasty (THA) has the potential advantage of being a single operative intervention with a single hospital stay, alongside reduced costs and total rehabilitation times [1, 2]. However, concerns over possible increases in perioperative complications, with studies reporting a higher incidence of surgical and medical complications versus unilateral or staged bilateral [3], have limited its use. In contrast, others have reported acceptable safety outcomes with sequential bilateral THA [3-5]. Most clinical investigations have demonstrated the safety of sequential bilateral THA in low-risk patients; *i.e*., with American Society of Anesthesiologists (ASA) category 1 or 2 disease [4, 6]. Approximately 10% of patients require contralateral THA within a year of their first THA operation [5].Anterior minimally invasive surgery (AMIS) for THA has been reported to be a safe and reliable surgical technique [7-10]. Advantages of simultaneous bilateral THA have been described for several surgical approaches [4, 6, 11-14]. However, there is a paucity of literature on simultaneous bilateral THA procedures using AMIS. The purpose of this retrospective study was therefore to assess whether sequential bilateral THA performed with an AMIS approach in appropriately selected patients is a safe and effective procedure. We hypothesized that sequential bilateral THA yields favorable clinical outcome and that it is safe to perform.

## 2 METHODS

Two surgical centers in Germany conducted an observational retrospective analysis of patients who underwent sequential bilateral THA using AMIS between June 2011 and December 2014. During the study period, 1.190 patients underwent THA, of whom 130 received simultaneous bilateral THA. Inclusion criteria for simultaneous bilateral THA were ASA categories 1, 2 and 3 bilateral hip disease. Exclusion criteria were general contraindications for surgery, a history of hip arthrodesis, ASA category 4 disease or greater, and revision surgery.Baseline Hb levels were optimized using iron supplementation and erythropoietin (EPO) if Hb level was ≤ 14.0 g/dl. In addition, postoperative iron therapy to compensate the intraoperative blood loss was provided, and we made use of a cellsaver for the reinfusion of autologous blood [15, 16]. The latter was performed as an immediate autologous blood transfusion postoperatively, which is associated with a significant decrease in the need for homologous blood transfusion [17]. All patients received 5 - 10 ml topical tranexamic acid prior to wound closure. Ninety-five patients were diagnosed with primary hip osteoarthritis, while 20 had sequelae of congenital hip dysplasia, 10 patients had rapidly destructive hip disease, and 5 had rheumatoid juvenile arthritis. Of the 130 patients, 77 (59.2%) were female and 53 (40.8%) male. Thirty-six (27.7%) were classified as ASA category 1, 93 (71.5%) as ASA category 2 and 1 (0.8%) as ASA category 3. The mean (± SD) age of the patients was 57.2 ± 7.3 years (range, 35-77 years).

All procedures were performed by the same surgical team, with the same lead surgeon. The procedures were performed consecutively in a two-session surgery while the patient was under anesthesia, using two instrument trays. A 20-minute break was given between the two sessions.

Patients received either a QUADRA or an AMIStem femoral component (Medacta, Castel San Pietro, Switzerland), which were combined with a Versafitcup (Medacta) and a ceramic femoral head (CeramTec, Plochingen, Germany). The operation was performed using AMIS as specified in the manufacturer’s instructions for use. The approach to the hip joint was done by an approximately 10 cm anterior incision, starting approximately 2 cm lateral and 1 cm distal to the anterior superior iliac spine and proceeding in a distal and slightly posterior direction. The incision was made laterally to the interval between the m. tensor fascia latae and the m. sartorius to minimize the risk for lateral femoral cutaneous nerve (LCFN) damage. By developing the interval within the tensor fascia, the LCFN is avoided in the superficial dissection. The facia is incised over the tensor and the medial side of the muscle to access the hip capsule. The hip capsule was opened anteriorly, retractors were placed medially and laterally of the capsule and the joint was dislocated. The acetabulum was reamed and the cup was inserted. Femoral exposure was enhanced by a trochanteric hook to elevate the proximal femur, and possibly by use of a fracture . Release of the lateral capsule from the medial greater trochanter allowed further femoral displacement anteriorly and laterally to facilitate broaching, femoral osteotomy and prosthesis insertion. A trial reduction of the joint was usually performed to check leg lengths and offsets. When the components were inserted, joint reduction and routine wound closure were done.

General anesthesia was used in all cases. None suction drain was placed in hip and all patients received prophylactic antibiotic therapy, as well as thromboembolism prevention with low-molecular-weight heparin for 4 weeks postoperatively. All 130 bilateral THA patients were invited to participate in the retrospective analysis and were sent a patient information sheet containing a detailed description of the study guidelines in June 2012. Participation in the study was voluntary and patients were allowed to inform their primary care physician about the study. Patients were asked to sign an informed consent form and told they could withdraw from the study at any time. None of the patients declined. For each patient, the following data were collected: total time of surgery; ASA score; intraoperative blood loss; length of hospital stay; number of homologous or allogeneic blood transfusions and complications, such as infection, dislocation, pulmonary embolism, thrombosis, or leg length discrepancy. For all patients, Hb level was determined by blood draws preoperatively and exactly 24 hours postoperatively. Blood loss was estimated during the surgical procedure by observing the aspirated amount of blood. Blood transfusion was administered at a Hb threshold of 8 g/dl, or as needed in patients who were symptomatic from their anemia. Functional outcomes were assessed in accordance with our standard follow-up protocol. Primary study outcome was the Harris Hip Score (HHS) [18], which was obtained preoperatively and at 6 weeks, 6 months, 12 months and 24 months postoperatively. Secondary outcomes were the High Activity Arthroplasty Score (HAAS) [19] and a subjective evaluation using the Questions on Life Satisfaction (FLZ) were assessed preoperatively, and at 6 months, 12 months and 24 months postoperatively [20]. We also documented mortality and morbidity rates following the procedure.The HHS can range from 0 to 100, with 100 representing the best possible outcome [18]. The HAAS can range from 0 to 18, where 0 is the worst possible score indicating severe symptoms and poor function of the joint, and 18 is the best score indicating excellent joint function [19]. The FLZ consists of two subscores: the “general life satisfaction score” and the “satisfaction with health score”. Minimum and maximum values for both scores are 16 and 80 [20]. Anteroposterior pelvic radiograph and two Lequesne false-profile radiographs were taken preoperatively and postoperatively for each patient.In accordance with German law, ethics committee approval was not obtained, as the study was purely observational, without any changes to standard clinical practice. The data were collected in an Excel spreadsheet (Microsoft, Redmond, WA) and were statistically analysed using SPSS for Windows (version 18.0, SPSS Inc. Chicago, IL, USA). The dependent sample t-test was applied. P <0.05 was considered statistically significant.

## 3 RESULTS

**Fig. (1):**
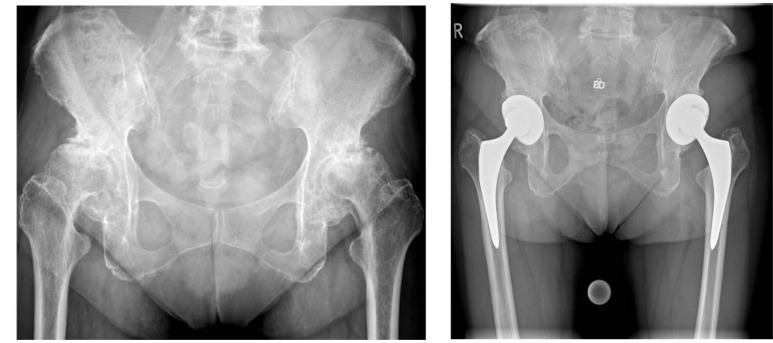
A 69 year old female patient. a) Preoperative anteroposterior radiograph, showing bilateral osteoarthritis. b) Immediate postoperative radiograph. Correct position of the implants.

The mean length of hospital stay was 7.4 ± 2.8 days (range, 5-18 days). Total mean duration of the procedure, including the lag time between the 2 procedures, was 161 ± 36 minutes (range, 92-260 minutes). The mean intraoperative blood loss was 518 ± 144 ml (range, 150 - 900 ml), and the mean hemoglobin level preoperatively and on the first postoperative day were 14.3 ± 0.7 g/dl (range, 12.5 - 17.3 g/dl) and 11.3 ± 1.1 g/dl (range, 7.3 - 13.9), respectively. Thirteen patients (10.0%) received an autologous blood transfusion, while 7 patients (5.4%) received one unit (800 ml) of allogeneic blood. No perioperative complications or deaths were recorded.One patient experienced a Vancouver B periprosthetic femoral fracture due to a fall 12 weeks postoperatively. The fracture was treated surgically with a cerclage, but the patient experienced a second traumatic periprosthetic femoral fracture 16 months postoperatively, after which he received a plate osteosynthesis. This patient was not included in the follow-up analysis. The HHS improved from 43.6 ±12.3 (range, 9 - 70) preoperatively to 98.8 ± 1.2 at final follow-up ( **1**). The HAAS improved from 7.1 ± 2.1 (range, 2 - 15) preoperatively to 14.0 ± 1.3 (range, 11 - 17) at 24 months. The general FLZ improved from 56.2 ± 6.7 (range, 44 - 79) to 68.4 ± 4.8 (range, 56 - 88), while the health FLZ improved from 58.8 ± 5.9 to 70.3 ± 4.6 (range, 60 - 87). The radiological assessment revealed that all femoral and all acetabular components were stable (Fig. **1**).

## 4 DISCUSSION

The favorable outcomes with sequential bilateral THA using the AMIS approach suggest that it is a viable alternative to two-stage bilateral THA in selected patients. In the present study, it was shown that bilateral simultaneous THA using an AMIS approach yields favorable clinical outcomes, with HHS and HAAS scores improving continuously up to 2 years postoperatively. The subjective satisfaction of patients, as measured on FLZ scores, was also high. Moreover, no complications attributable to the procedure were observed. There were also no deaths in our population. The latter finding is consistent with the literature. The risk of death after sequential bilateral THA is no higher than the cumulative risk associated with two-stage bilateral THA [12, 16, 21]. As has been shown in previous studies, sequential bilateral THA decreases costs [1] and shortens rehabilitation time [11]. Better postoperative outcomes after sequential bilateral THA than after two-stage bilateral THA have been reported in several patient series [22, 23], and have been confirmed in a meta-analysis [12]. With regard to safety, the study findings are in concordance with a recent meta-analysis that concluded that simultaneous bilateral THA does not lead to an unacceptably high rate of complications or rate of death, compared with a staged bilateral approach [12].Although no control group was employed in the present study, the favorable outcomes and the absence of procedure-related complications may suggest that findings from these previous studies can be generalized to the bilateral THA using an AMIS approach. It is important that a blood-sparing strategy is used for bilateral THA. Most of the studies reported increases in homologous blood transfusion rates of 20% to 40% after sequential THA [24]. The mean LOS found in the present study was 7.6 days, which is above the average as reported in the literature [25-27]. Germany has globally one of the longest duration of stay in the hospital. Between-country variation can be largely explained by different treatment patterns and care organization, including arrangements about postoperative rehabilitation [25]. It must be noted that LOS for unilateral procedures during the same time interval in our hospital was 5.1 days, which is significantly lower than for bilateral procedures. This finding is consistent with the literature [12, 27]. The strength of the study is the prospective documentation of outcomes, alongside the low rate of attrition. The limitations of the study are its retrospective design, the lack of a control group, and the absence of a-priori study hypothesis. The study was not adequately powered to allow for definitive inferences on the safety of the procedure. Another limitation was the liberal eligibility criteria with regard to comorbidity status. ASA category 3 patients were also eligible for the study. The literature indicates that sequential bilateral THA should be reserved for ASA categories 1 and 2, as the complication rate is high in ASA category 3 patients, who have a threefold higher relative risk of death [28]. A single case with ASA 3 was included in the present study, and therefore this study does not add to the body of knowledge with regard to the outcome in this patient cohort. The patient who experienced a periprosthetic fracture was not included in the follow-up assessments, which biases study outcomes. A final limitation was that all procedures were performed by a single surgeon and the same surgical team, who conduct a high volume of surgeries annually. Consequently, the findings of the present study are not readily generalizable to other clinical settings. Inferences should therefore be drawn with caution, and larger multicenter, prospective, controlled studies will be required.

## CONCLUSION

In conclusion, our results support the use of sequential bilateral THA in carefully selected patients, particularly as no complications were reported, but further studies are needed to confirm these findings.

## Figures and Tables

**Table 1:** Clinical results.

–	–	Follow-up			
–	Preoperative	6 Weeks	6 Months	12 Months	24 Months
Harris Hip Score	44.6 ± 13.1	78.1 ± 8.7	95.4 ± 2.2	98.2 ± 1.4	98.9 ± 1.1
HAAS	7.5 ± 2.3		12.3 ± 1.5	13.7 ± 1.4	14.3 ± 1.0
FLZ - General - Health	57.7 ± 6.6 59.6 ± 6.1	N.r. N.r.	62.8 ± 6.3 66.4 ± 5.0	64.8 ± 6.1 67.1 ± 10.3	68.6 ± 5.1 70.9 ± 70.9

Presented as mean ± standard deviation. Abbreviations: Haas, High Activity Arthroplasty Score; FLZ, Questions on Life Satisfaction; N.r., not recorded
